# Evaluation of Three Feasibility Tools for Identifying Patient Data and Biospecimen Availability: Comparative Usability Study

**DOI:** 10.2196/25531

**Published:** 2021-07-21

**Authors:** Christina Schüttler, Hans-Ulrich Prokosch, Martin Sedlmayr, Brita Sedlmayr

**Affiliations:** 1 Chair of Medical Informatics Friedrich-Alexander-Universität Erlangen-Nürnberg Erlangen Germany; 2 Institute for Medical Informatics and Biometry Carl Gustav Carus Faculty of Medicine Technische Universität Dresden Dresden Germany

**Keywords:** software tools, user interface, feasibility, evaluation, research

## Abstract

**Background:**

To meet the growing importance of real-word data analysis, clinical data and biosamples must be timely made available. Feasibility platforms are often the first contact point for determining the availability of such data for specific research questions. Therefore, a user-friendly interface should be provided to enable access to this information easily. The German Medical Informatics Initiative also aims to establish such a platform for its infrastructure. Although some of these platforms are actively used, their tools still have limitations. Consequently, the Medical Informatics Initiative consortium MIRACUM (Medical Informatics in Research and Care in University Medicine) committed itself to analyzing the pros and cons of existing solutions and to designing an optimized graphical feasibility user interface.

**Objective:**

The aim of this study is to identify the system that is most user-friendly and thus forms the best basis for developing a harmonized tool. To achieve this goal, we carried out a comparative usability evaluation of existing tools used by researchers acting as end users.

**Methods:**

The evaluation included three preselected search tools and was conducted as a qualitative exploratory study with a randomized design over a period of 6 weeks. The tools in question were the MIRACUM i2b2 (Informatics for Integrating Biology and the Bedside) feasibility platform, OHDSI’s (Observational Health Data Sciences and Informatics) ATLAS, and the Sample Locator of the German Biobank Alliance. The evaluation was conducted in the form of a web-based usability test (usability walkthrough combined with a web-based questionnaire) with participants aged between 26 and 63 years who work as medical doctors.

**Results:**

In total, 17 study participants evaluated the three tools. The overall evaluation of usability, which was based on the System Usability Scale, showed that the Sample Locator, with a mean System Usability Scale score of 77.03 (SD 20.62), was significantly superior to the other two tools (Wilcoxon test; Sample Locator vs i2b2: *P*=.047; Sample Locator vs ATLAS: *P*=.001). i2b2, with a score of 59.83 (SD 25.36), performed significantly better than ATLAS, which had a score of 27.81 (SD 21.79; Wilcoxon test; i2b2 vs ATLAS: *P*=.005). The analysis of the material generated by the usability walkthrough method confirmed these findings. ATLAS caused the most usability problems (n=66), followed by i2b2 (n=48) and the Sample Locator (n=22). Moreover, the Sample Locator achieved the highest ratings with respect to additional questions regarding satisfaction with the tools.

**Conclusions:**

This study provides data to develop a suitable basis for the selection of a harmonized tool for feasibility studies via concrete evaluation and a comparison of the usability of three different types of query builders. The feedback obtained from the participants during the usability test made it possible to identify user problems and positive design aspects of the individual tools and compare them qualitatively.

## Introduction

Real-world data analysis in medicine is becoming increasingly important and relies on the timely availability of clinical data and biosamples collected during clinical care processes in university hospitals [1,2]. The exploitation and use of such data are two of the major goals of several national and international initiatives [3-5]. In Germany, this goal is being pursued with a nationwide approach, particularly through the Medical Informatics Initiative (MII), in which all university hospitals have joined forces in four consortia [6]. However, a crucial aspect of this process is not only the allocation and preparation of data from the respective source systems but also their findability for external interest groups such as researchers. For this purpose, the feasibility platforms are a common first contact point before writing a data use request and submitting it to the data provider. At this level, researchers can initially verify whether the affiliated institution has a suitable number of patient records for a planned research project. This usually requires a graphical user interface that can formulate a description of the desired patient cohort based on the study criteria. The MII also aims to establish such a platform as part of its central portal. Although there are various projects that have already implemented such a platform, the tools used have specific limitations, such as single source compatibility, a reduced number of temporal constraints available [7], and limited usability [8]. Consequently, MIRACUM (Medical Informatics in Research and Care in University Medicine) [9], one of the four MII consortia, has set itself the task of carrying out a comparative evaluation of existing tools with regard to their usability to identify the most user-friendly system, and thus forms the best basis for developing a harmonized tool. As the implementation of such a platform was intended to take place as quickly as possible, a preselection of three implementations already used in the consortium (also freely accessible for researchers in Germany) was made for the usability evaluation: the MIRACUM i2b2 (Informatics for Integrating Biology and the Bedside) [10] feasibility platform, OHDSI’s (Observational Health Data Sciences and Informatics) ATLAS [11,12], and the Sample Locator [13] of the German Biobank Alliance (GBA) [14,15]. The selection was based on the fact that they differed greatly in terms of complexity and functionality, so good coverage was expected. The usability analysis focused on two questions: (1) which tool offers the best usability (overall) and hence forms the most suitable foundation for creating a balanced tool? and (2) in which areas and with regard to which usability aspects are the tools rated better or worse in comparison and which recommendations can be derived for further development?

This paper describes the procedure used to answer these research questions. As the focus was on the evaluation of user-friendliness, a usability analysis was conducted with potential end users—laypeople, who should be enabled to conduct feasibility studies. To the best of our knowledge, there has not yet been a study comparing these three query builders—i2b2, ATLAS, and the Sample Locator—in terms of usability. This study should address this gap in the scientific literature. The methodological approach in this study can serve as a model for decision makers and researchers of similar projects. The insights gained from the evaluation of the tools by clinically active researchers will subsequently be used for the further development of a unified tool.

## Methods

### Study Design

To evaluate the previously selected search tools, a qualitative exploratory study with a randomized design over a period of 6 weeks (from August 3, 2020, to September 13, 2020) was conducted. It was carried out in the form of a web-based usability test (usability walkthrough combined with a web-based questionnaire) with female and male participants aged between 26 and 63 years who work as medical doctors that are also engaged in research. In advance of this study, ethical approval was obtained from the Technical University of Dresden (Germany) ethics committee (SR-EK-262062020).

### Recruitment

For a valid evaluation of usability, the study concept called for a study size of 30 subjects. This corresponds to three researchers per MIRACUM site (n=10). Given the number of test persons, it can be assumed that the majority of all usability problems are discovered [16]. A contact person at the respective location identified and approached suitable study participants. In addition to the requirement of being clinically active and engaged in research, the test participants were required to have no experience with the tools to be evaluated, enough time to test all systems and answer a questionnaire, and be willing to record the test. In case of interest in participating in the study, the contact details were forwarded to the study team. At the start of the study, the participants received an email containing all relevant documents for conducting the evaluation. In addition, the study information and a consent form were attached, which needed to be signed and returned to the study team after completion of the study.

### Material

**Figure 1:**
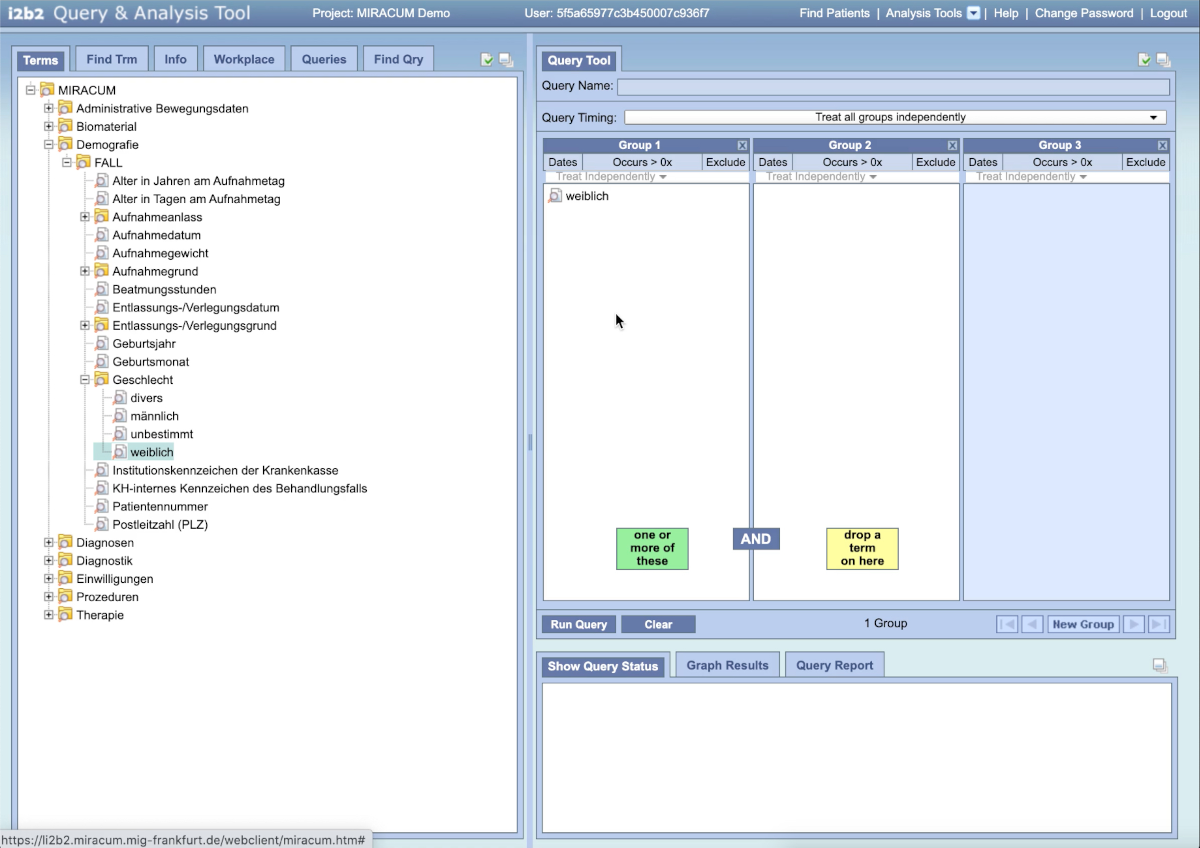
Example of a query built using the MIRACUM i2b2. On the right side of the screen, the user can select the appropriate parameters and then drag and drop them into “AND-linked" groups on the left side of the screen. Exclusion criteria are defined by the “Exclude” option in the groups. The search is executed by selecting the button “Run Query.” i2b2: Informatics for Integrating Biology and the Bedside; MIRACUM: Medical Informatics in Research and Care in University Medicine.

**Figure 2:**
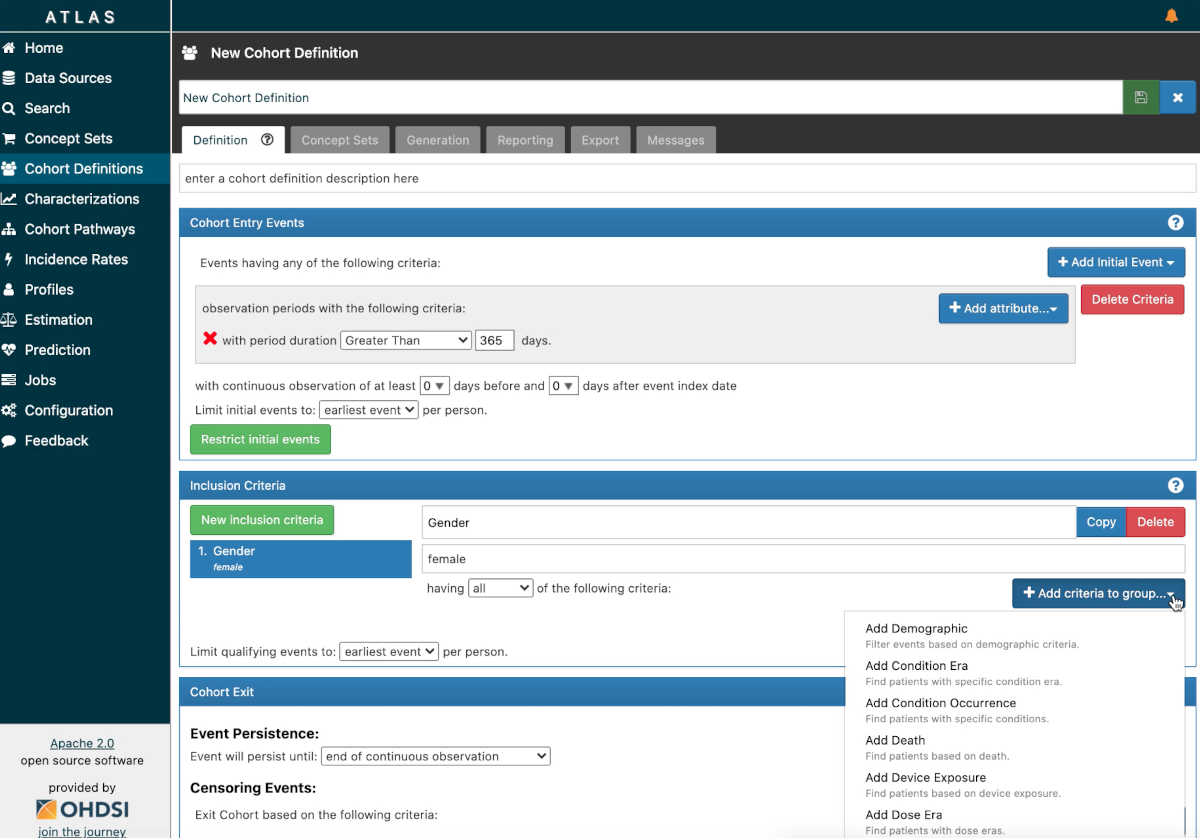
Example of a query built using OHDSI’s ATLAS. The criteria in the form of concepts can be selected and linked by selecting the “New Inclusion Criteria” button. The definition of an exclusion criterion is made by defining it as a “noninclusion.” ATLAS requires that an entry and exit event must be defined for the search. The search is executed via the “Generation” tab. OHDSI: Observational Health Data Sciences and Informatics.

**Figure 3:**
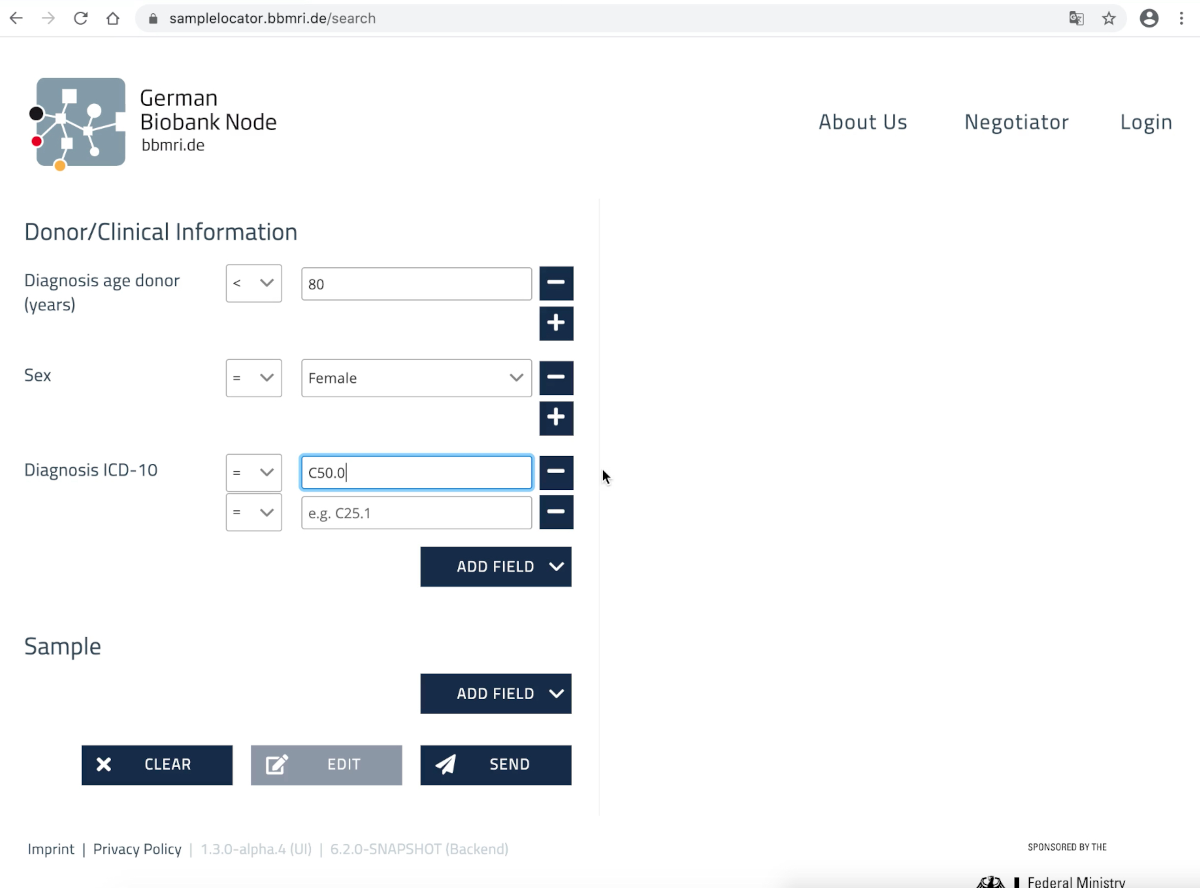
Example of a query built using the German Biobank Alliance Sample Locator. With the Sample Locator, the corresponding criteria are compiled via the selection menus. Input fields within a criterion (eg, diagnosis, as shown in the figure) are linked with “OR,” and input fields between criterion fields are linked with “AND.” An exclusion can be defined using the operator “not equal to.” The search is executed by selecting the “Send” button.

The three tools to be evaluated are the MIRACUM i2b2 feasibility platform (webclient version 1.7.12), OHDSI’s ATLAS (version 2.7.7/2.7.8), and GBA’s Sample Locator (user interface version 1.3.0-alpha.4 and backend version 6.2.0). The MIRACUM i2b2 feasibility platform is based on proprietary (but internationally widely used) data structures. It is currently based on the six basic modules of the MII core data set and supports participation in international large-scale research [17] (Figure 1). ATLAS is primarily a web interface that allows the use of various OHDSI tools. Functionalities include search and navigation within the OMOP (Observational Medical Outcomes Partnership) Common Data Model Vocabulary database to identify patient cohorts (Figure 2). The third tool is the Sample Locator, which is designed to search for samples and related data from GBA-affiliated biobanks (Figure 3). Although the i2b2 and OHDSI ATLAS clients are already heavily applied in international data sharing networks [10,11], the GBA Sample Locator is productive as a first version.

The usability analysis of the examined tools was based on the processing of three tasks. The tasks were structured in such a way that they increased in complexity. Although the first task only required the selection of inclusion criteria (gender, diagnosis, therapy, and laboratory test), the following task also asked for a parameter to be defined as an exclusion criterion. The final task included a time component, which was queried by specifying a diagnosis period. For the sake of comparability, the respective tasks were coordinated accordingly between the tools, taking into account the tool-specific functionalities (Table 1).

**Table 1:** Queries construction. The users were asked to construct a query according to these specified criteria and find the number of corresponding patients or biosamples.

Criterion type and criterion				i2b2^a^		Criterion		ATLAS		Criterion		Sample locator
**Query 1**												
	Cohort entry event			N/A^b^		Observation period		Duration: >365 days		N/A		N/A
	**Inclusion**											
		Gender	Female		Gender		Female		Sex		Female	
		Diagnosis	Malignant neoplasm of the brain		Condition occurrence		Malignant neoplasm of the brain		Diagnosis		Carcinoma mammae	
		Treatment	Temozolomide		Treatment		Temozolomide		Age		<80 years	
		Lab values	Platelet count: <50.000/uL		Lab values		Platelet count: <50.000/uL		Sample type		Tissue stored in formalin	
	Cohort exit			N/A		Event will persist until:		End of continuous observation		N/A		N/A
**Query 2**												
	Cohort entry			N/A		Observation Period		Duration: >365 days		N/A		N/A
	**Inclusion**											
		Age	>18 years		Age		>18 years		Sex		Male	
		Diagnosis	Type 2 diabetes mellitus		Diagnosis		Type 2 diabetes mellitus		Diagnosis		Atherosclerotic cardiovascular disease	
		Lab values	Hemoglobin between 13 and 18 g/dL		Lab values		Hemoglobin between 13 and 18 g/dL		Biosamples		Serum, storage temperature: −70°C OR^c^ plasma stabilized, storage temperature: −70°C	
	**Exclusion**											
		Diagnosis	Myocardial infarction		Diagnosis		Myocardial infarction		N/A		N/A	
	Cohort exit			N/A		Event will persist until:		End of continuous observation		N/A		N/A
**Query 3**												
	Cohort entry			N/A		Observation Period		Duration: >365 days		N/A		N/A
	**Inclusion**											
		Age	>65 years		Age		>65 years		Age		<18 years	
		Diagnosis	Essential (primary) hypertension		Diagnosis		Hypertensive disease		Diagnosis		Thyroid nodule	
		Biosamples	Serum		Lab values		LDL^d^ cholesterol measurement: value >200		Biosamples		Tissue snap frozen	
	**Temporal constraints**											
		Diagnosis period	Between 01/01/2020 and 04/30/2020		Diagnosis period		Between 01/01/2020 and 04/30/2020		Diagnosis period		Between 01/01/2020 and 04/30/2020	
	**Exclusion**											
		Treatment	Lipid-lowering drugs		Treatment		Lipid-lowering drugs		Diagnosis		Concurrent diagnosis of thyroid cancer	
	Cohort exit			N/A		Event will persist until:		End of continuous observation		N/A		N/A

^a^i2b2: Informatics for Integrating Biology and the Bedside.

^b^N/A: not applicable; the criterion is not applicable for this tool.

^c^The task was to include this criterion with an OR operator.

^d^LDL: low-density lipoprotein.

During the task processing, the participants were asked to record their interactions on video and to express their thoughts (what causes them difficulties and what they like about the system) aloud (so-called *Thinking-Aloud* method) [18]. With the help of the screen recordings as well as the comments of the participants, which were made during the processing of the test, usability problems could be identified and positive or negative aspects of the interaction could be detected.

In addition, a web-based questionnaire was developed for the final assessment of usability. This questionnaire consisted of the following four parts (parts A-D):

- Parts A-C: three question blocks for assessing the usability of each query builder based on the (standardized) System Usability Scale (SUS) [19] and self-developed questions about satisfaction
- Part D: a final question block for a comparative rating of the query builders and for collecting demographic information (eg, age, gender, work experience, previous experience with queries and similar systems, and computer expertise).

The SUS questions and the supplementary questions on satisfaction were to be rated on a five-level rating scale (strongly disagree, disagree, neither agree nor disagree, agree, or strongly agree). For the questions about the person, the corresponding answer options had to be selected or certain blanks had to be filled in.

All test tasks and the web-based questionnaire were pretested in advance. A complete version of the questionnaire is provided in Multimedia Appendix 1.

### Study Flow

The study material included an individualized test manual. It provided the framework and contained all the steps that needed to be taken to successfully conduct the study. The test was designed as an individual session at the workplace of the person (or alternatively in the home office), with a duration of approximately 90 minutes. As the harmonized tool to be developed should primarily address laypeople or casual users and as the evaluation focused on intuitive use, self-descriptiveness, and easy learnability of existing query builders, no training was conducted in advance with the participants. First, the participants were asked to install the screen recording software according to the instructions provided. Once the technology was established, the test subjects evaluated all tools while working through the respective test tasks (Table 1). The order in which the systems were to be tested was randomized to avoid bias caused by learning effects. The sequence of actions was recorded during the execution of the test tasks. In addition, the testers were asked to verbalize their thoughts about their individual steps in processing. After completion of the task complex of one tool, the subjects were asked to answer related usability and satisfaction questions immediately before continuing with the next system. When the test users encountered difficulties in accomplishing the tasks, the study material contained a rudimentary guide on how to use the tools. Finally, questions about the final and comparative ranking of the tools and about the person (demographic information) were answered. Once the usability test was completed, the screen recording files had to be loaded into a secured cloud storage by each test subject.

### Data Analysis

#### Analysis of Screen Recordings (Interactions and Expressions)

The statements and actions recorded on the screen videos of all test persons were transcribed per system by 2 members of the study team. The protocols were subsequently mutually validated. The transcripts were then scanned by these 2 members for negative aspects or problem areas and positive aspects. Subsequently, all problems or positive statements were collected in an overall list (ie, if a problem was named several times by different test subjects or if it occurred across all test tasks, it was noted as one problem). Each problem was evaluated and rated by 2 independent raters in terms of its severity according to the Nielsen and Mack [20] severity rating, ranging from 0 (no usability problem) to 4 (usability catastrophe). Rating differences between the 2 evaluators were discussed until a consensus was reached.

Furthermore, the problems were classified according to Zapf error taxonomy [21] into use problems (resulting from a lack of fit between user and software) or functional problems (incomplete or missing functionality of a system), as follows:

- Examples of use problems: errors of knowledge, errors of thinking, errors of memory and forgetting, errors of judgment, errors of habit, errors of omission, errors of recognition, and errors of movement
- Examples of functional problems: action blockades, action repetitions, action interruptions, and alternative course of action.

In addition, videos were used to determine how successfully the respective test person completed the tasks. A test task was considered correct if all parameters were entered and if they were correctly linked in the system. A task was considered incorrect if the parameters were incomplete, the link between the parameters was incorrect, or both situations occurred.

#### Analysis of the Web-Based Questionnaire (Usability and Satisfaction Ratings and Demographic Information)

The questions of the SUS were analyzed using the scoring method by Brooke [19], which yields possible values from 0 to 100 and allows the values to be compared with the values of a grading scale, where 0 represents an unacceptable usability and 100 represents the best imaginable usability. The additionally formulated questions on satisfaction with the query builder were converted into a numerical scale ranging from 1 (strongly disagree) to 5 (strongly agree). For descriptive analysis, mean scores and SDs were calculated. For the demographic questions (depending on the question type), the percentage was calculated, mean values and SDs were determined, or the free text was analyzed. For open-ended answers (free text), thematic categories were defined, and the answers of the test persons were assigned to these categories. Cases with missing values were deleted from the list. The Wilcoxon rank sum test was used to statistically compare the questionnaire results between the three query builders. The Pearson correlation was calculated to analyze whether demographic variables had an influence on the evaluation results. The significance level was set at *P*<.05. All statistical analyses were performed using SPSS 27.0 (IBM Corporation).

## Results

### Participant Characteristics

Of the 30 potential study participants, 17 (57%) responded. Due to the early termination of the study and the testing of only one query builder, 1 participant had to be excluded. Thus, the data from 16 participants were analyzed. The participants had an average age of 38.13 years (SD 9.68) and about two-thirds of the subjects were male (10/16, 63%). The average work experience was 10.37 years (SD 10.86). The majority of the participants worked in clinical research or as research assistants (13/16, 81%), whereas 2 participants assigned themselves to other professional groups (professor and quality manager). As far as computer skills are concerned, everyone rated themselves well; either they said that they could handle most systems properly (8/16, 50%) or that they had a significant amount of experience and were technically proficient (7/16, 44%). Only 3 persons stated having previous experience with systems similar to those tested in the usability evaluation (3/16, 19%). In general, less than half of the respondents stated that they had general experience with requesting case numbers for clinical studies (little experience: 5/16, 31% or a lot of experience: 2/16, 13%). The full sample characteristics are presented in Table 2.

**Table 2:** Characteristics of the participants (N=16). Summarized number and percentage per category. For age and work experience mean and SD were calculated.

Variable				Values
**Age**				
	**Answered, n (%)**			15 (94)
		Age (years), mean (SD)	38.13 (9.680)	
	No answer, n (%)			1 (6)
**Gender, n (%)**				
	Male			10 (63)
	Female			5 (31)
	No answer			1 (6)
**Native language, n (%)**				
	German			14 (88)
	Other: Hungarian			1 (6)
	No answer			1 (6)
**Difficulties regarding English, n (%)**				
	Never			7 (44)
	Rarely			7 (44)
	Sometimes			1 (6)
	No answer			1 (6)
**Professional group, n (%)**				
	Clinical researcher			6 (38)
	Scientific assistant			7 (44)
	Other: professor or quality manager			2 (12)
	No answer			1 (6)
**Work experience**				
	**Answered, n (%)**			13 (81)
		Work experience (years), mean (SD)	10.37 (10.861)	
	No answer, n (%)			3 (19)
**Experience with feasibility studies, n (%)**				
	No experience or little experience			8 (50)
	Some experience			5 (31)
	Much experience			2 (13)
	No answer			1 (6)
**Use of similar systems in the past, n (%)**				
	No			12 (75)
	Yes			3 (18)
	No answer			1 (6)
**Computer skills, n (%)**				
	Average computer skills			8 (50)
	Excellent computer skills			7 (44)
	No answer			1 (6)

### Think-Aloud Test Results

#### Negative and Positive Design Aspects

The evaluation of the material generated by the *Thinking-Aloud* method revealed concrete usability problems. Classification according to the severity scale produced the following result: ATLAS had the most usability problems, with 66 problems noted. These were divided into 21 major problems, 30 minor problems, and 15 cosmetic problems. A major problem was the function for saving:

That’s where it starts: How and where to save? I don’t know. Where is it stored here? I have no idea.

With i2b2, the 48 detected problems were divided into 9 major, 26 minor, and 13 cosmetic problems. Among other things, it was noted that the procedure for defining an exclusion criterion is not clear. The Sample Locator had the lowest number of problems, with 22 problems noted. In contrast to the other tools, however, there are also two problems with the level usability catastrophe. Furthermore, 4 major, 10 minor, and 6 cosmetic problems were identified. One of the usability disasters concerned the *AND* or *OR* combination of the individual criteria. The logic behind this was often not obvious to the users, which became apparent from their comments:

The question is, how do you represent this “OR” connection here. This is not quite clear now.

So, a bit unclear to be honest, whether this is “AND” or “OR.”

In addition to the critical aspects, some positive points and suggestions for improvement could be extracted. In the case of ATLAS, a large number of possible options and settings were highlighted as positive. The same applies to the visualization of the results, which are displayed in the form of a colored square with subareas for the selected criteria. As starting points for the improvement of the handling of a suggestion list for the input of criteria, the specification of units as well as the plausibility check before the start of the search was mentioned. i2b2 was able to convince with its intuitive operating concept, where the elements can be easily assigned to the groups via the drag-and-drop function. In addition, the automatically appearing input window for values, as soon as a criterion was selected, was considered supportive. However, it should be possible to select criteria not only from an ontology tree but also via a free-text search. With the Sample Locator, a more comprehensive arrangement of the criteria was desired. For example, the division into *Donor and Clinical Information* and *Sample* and their meaning was not immediately obvious, and the order of the individual criteria could also be improved, for example, thematically related criteria were placed one below the other. However, the Sample Locator was found to be very clear, straightforward, and intuitive to use, so the tasks were “nice and also very easy to implement”. Multimedia Appendix 2 shows the most serious usability problems (severity ratings of 3 and 4) of the respective query builders with the corresponding number of participants who have named this problem and the resulting optimization recommendations.

#### Task Success

**Figure 4:**
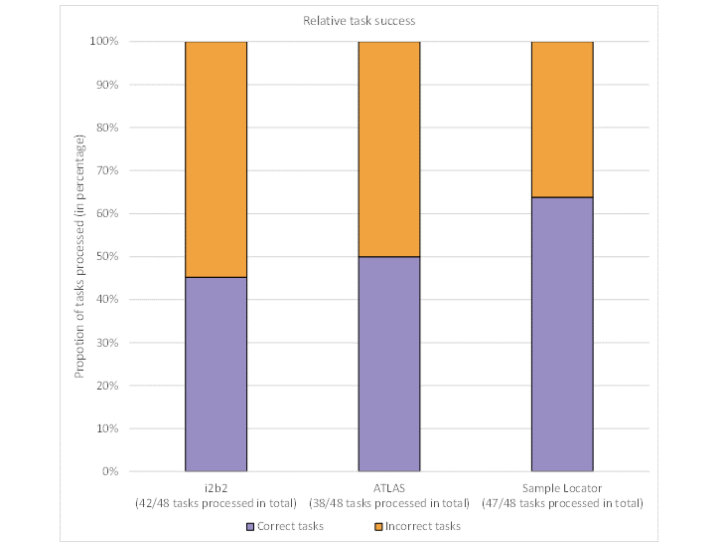
Relative task success for the three query builders. Success was denoted when all required parameters were entered and linked correctly. i2b2: Informatics for Integrating Biology and the Bedside.

Of the 48 tasks evaluated per tool, 47 were completed in Sample Locator, with 30 of them processed correctly and 17 of them not processed correctly. Nineteen tasks were completed successfully for both ATLAS and i2b2. On the contrary, 19 and 23 tasks could not be executed correctly with ATLAS and i2b2, respectively. In the case of i2b2, 6 tasks were not processed, and in the case of ATLAS, 10 tasks were missing. Overall, the Sample Locator scored the best overall test items in terms of absolute correctness. Considering the correctness of the task processing relative to all finished tasks across all respondents, there was no significant difference between the query builders (Wilcoxon test; i2b2 vs Sample Locator: *P*=.07; i2b2 vs ATLAS: *P*=.72; ATLAS vs Sample Locator: *P*=.06). The false or unprocessed tasks were relatively evenly distributed among the three tasks in i2b2 and ATLAS (Figure 4).

### Questionnaire Results

#### Results of the SUS

The overall evaluation of usability based on the SUS showed that the Sample Locator, with a mean SUS score of 77.03 (SD 20.62), was significantly superior to the other two tools (Wilcoxon test; Sample Locator vs i2b2: *P*=.047; Sample Locator vs ATLAS: *P*=.001). However, i2b2, with a score of 59.83 (SD 25.36), still performed significantly better than ATLAS, which had a score of 27.81 (SD 21.79; Wilcoxon test; i2b2 vs ATLAS: *P*=.005). For reasons of comprehensibility (positive and negative usability aspects), the individual results of the SUS are presented in Table 3. However, only the overall SUS score can be interpreted as a measure of usability. Using the Pearson correlation, no association between SUS scores and the personal variables age (correlation age-SUS; i2b2: *P*=.87; ATLAS: *P*=.14; Sample Locator: *P*=.66), gender (correlation gender-SUS; i2b2: *P*=.44; ATLAS: *P*=.85; Sample Locator: *P*=.20), work experience (correlation work experience-SUS; i2b2: *P*=.95; ATLAS: *P*=.32; Sample Locator: *P*=.40), and previous experience with cohort research (correlation experience cohort research-SUS; i2b2: *P*=.70; ATLAS: *P*=.58; Sample Locator: *P*=.60) was evident.

**Table 3:** Results of the SUS^a^. Mean ratings from 1 (strongly agree) to 5 (strongly disagree) and SDs are presented.

SUS item	i2b2^b^ (n=15), mean (SD)	ATLAS (n=16), mean (SD)	Sample Locator (n=16), mean (SD)
I think that I would like to use this query builder frequently.	3.60 (1.242)	2.00 (1.000)	3.75 (1.125)
I found this query builder unnecessarily complex.^c^	3.33 (1.234)	1.75 (0.829)	4.44 (0.892)
I thought this query builder was easy to use.	3.13 (1.187)	1.94 (1.029)	4.31 (0.873)
I think that I would need the support of a technical person to be able to use this query builder.^c^	3.53 (1.125)	2.81 (1.333)	4.44 (0.727)
I found the various functions in this query builder were well integrated.	3.27 (1.223)	2.25 (1.031)	3.69 (1.138)
I thought there was too much inconsistency in this query builder.^c^	3.60 (0.737)	2.75 (1.090)	3.75 (1.000)
I would imagine that most people would learn to use this query builder very quickly.	3.27 (1.387)	1.69 (0.982)	4.06 (0.998)
I found this query builder very cumbersome to use.^c^	3.20 (1.424)	1.81 (0.882)	4.13 (1.408)
I felt very confident using this query builder.	3.33 (1.047)	1.88 (0.927)	3.75 (0.856)
I needed to learn a lot of things before I could get going with this query builder.^c^	3.67 (1.113)	2.31 (1.261)	4.50 (0.816)

^a^SUS: System Usability Scale.

^b^i2b2: Informatics for Integrating Biology and the Bedside.

^c^Reverse-coded item.

#### Results of the Additionally Formulated Questions on Satisfaction

The additional questions regarding the satisfaction with the tools confirm the outcome of the SUS. The Sample Locator achieves the highest ratings, with the exception of the subjectively perceived working speed, which was felt to be the least slowed down with i2b2. The test participants were also satisfied with i2b2, but the Sample Locator was rated significantly more positively with regard to the presentation of query results (Wilcoxon test; *P*=.03), the ability to undo operating steps (Wilcoxon test; *P*=.01), navigation within the tool (Wilcoxon test; *P*=.005), presentation of information (clarity; Wilcoxon test; *P*=.04), and visual design (Wilcoxon test; *P*=.02). For ATLAS, with the exception of the item *possibility of undoing task steps*, all ratings were generally in the negative range in every aspect of satisfaction (Table 4).

**Table 4:** Results of the satisfaction rating. Mean ratings from 1 (strongly agree) to 5 (strongly disagree) and SDs.

Satisfaction with the query builder	i2b2^a^ (n=15), mean (SD)	ATLAS (n=16), mean (SD)	Sample Locator (n=16), mean (SD)
I am satisfied with the ease with which the tasks can be accomplished.	3.20 (1.265)	1.81 (0.808)	4.06 (1.063)
I am satisfied with the time it takes to complete the tasks.	3.60 (1.242)	1.75 (1.031)	4.31 (0.793)
I am satisfied with the functionality that is provided to complete the tasks.	3.27 (1.280)	2.38 (1.317)	3.69 (1.014)
The terms and designations used in the query builder (eg, for the selection options and for patient characteristics) are immediately understandable to me.	4.00 (0.756)	2.19 (1.130)	4.25 (0.683)
The query builder enables me to complete work steps (eg, the selection of certain clinical or temporal parameters) in the order that seems to make the most sense to me.	3.53 (1.407)	2.88 (1.218)	3.88 (1.147)
The results generated with the query builder are displayed or output in such a way that they meet my requirements (eg, through clear grouping and an attractive visualization).	2.80 (1.265)	2.19 (1.073)	3.56 (1.094)
It is immediately apparent to me which consequences my input in the query builder has.	3.13 (1.302)	1.81 (0.882)	3.19 (1.223)
The query builder offers me the possibility to undo work steps if it is appropriate for my task completion.	3.93 (0.884)	3.88 (0.857)	4.63 (0.619)
I found the navigation within the query builder easy.	3.40 (1.242)	1.75 (0.901)	4.44 (0.892)
I found the information displayed in the query builder to be clear and concise.	3.13 (1.187)	1.81 (0.808)	4.00 (1.317)
The user interface of the query builder is visually appealing.	2.80 (1.424)	2.50 (1.118)	4.19 (1.223)
During my work with the query builder, errors occurred (eg, that options could not be combined and that exclusion criteria did not work).^b^	3.47 (1.552)	2.69 (1.102)	4.19 (1.047)
I sometimes felt slowed down in my work speed by the query builder (eg, by too long waiting times).^b^	4.00 (1.134)	2.63 (1.317)	3.69 (1.352)

^a^i2b2: Informatics for Integrating Biology and the Bedside.

^b^Reverse-coded item.

## Discussion

### Overview

The motivation for this study was to compare three different feasibility platforms and to answer the following questions: (1) which of the systems is best suited as a basis for further development and (2) which positive aspects can be taken over from the other tools for the purpose of more user-friendliness. This paper not only discusses the answers to these questions but also illustrates the approach to achieve this.

### Discussion of Methods

To answer the research questions, a web-based usability test with end users and the established usability methods *Thinking-Aloud* and the questionnaire based on the standardized SUS was chosen as the methodological design.

An advantage of web-based usability testing is the independence of time and place with which such tests can be performed, and no extra test or observation room is required. Web-based testing is a very time-efficient method that allows several people to test a system at the same time. However, this method also has disadvantages: observers have no real-time access to data, and there is no possibility of interacting with the user during data collection [22]. However, studies show that a remote test provides as valid results as a laboratory test: Tullis et al [23], for example, presented results that show high correlations between laboratory and remote tests for task completion data and task time data. The most critical usability problems were identified using both the techniques. In a study conducted by Andreasen et al [24], three methods for remote usability testing and a traditional laboratory-based *Thinking-Aloud* method were compared. These results also show that the remote method is equivalent to the traditional laboratory method. Therefore, our choice of a web-based test can be considered equivalent to a usability study conducted in the laboratory.

For our web-based usability test, we chose the methods *Thinking-Aloud* and questionnaires. The *Thinking-Aloud* method allowed us to find out what potential users actually think about the query builder. In particular, it enabled us to identify usability problems that could lead to feasible redesign recommendations. We learned why some parts of the user interface are difficult to use and which areas of the tools are easy and intuitive for the user. Advantages of this method are that no special equipment is required for this method, it does not take a lot of time, and data can be collected very quickly, which is sufficient for the most important insights. Furthermore, this method is independent of the level of technical experience of the test persons and can be used for any type of user interface. A disadvantage of the *Thinking-Aloud* method, however, is that it is generally not suitable for detailed statistics. In addition, the situation of constantly expressing thoughts is very unnatural, which makes it difficult for the test person to maintain the required monolog [25]. Thus, we could also observe that some of our test participants temporarily forgot to verbalize their thoughts. To compensate for the disadvantages of this method, we combined it with a questionnaire on usability and satisfaction with the query builders.

Questionnaires have the advantage that numerous data can be obtained with relatively little effort. The use of standardized questionnaires also supports the objectivity of data collection and allows comparisons between systems [26]. In particular, the SUS is a very reliable questionnaire that detects differences even in small sample sizes. In addition, it has been shown that SUS can effectively distinguish between systems with low and high usability and also correlates to a high degree with other questionnaire-based usability measurement tools [27]. However, questionnaires such as SUS are not suitable for diagnosing usability problems and gathering background information to understand why users evaluate a system in this way. In addition, the relevant aspects of the given questions may be lost. However, we were able to compensate for this disadvantage by using the *Thinking-Aloud* method in combination.

In summary, by combining different methods, the advantages and disadvantages of the respective methods can be balanced and a comprehensive opinion can be obtained. According to Sarodnick and Brau [28], the combination of observation and spontaneous expression of thoughts ensures a high validity of the data.

### Discussion of Results

Our first research question should answer which of the three query builders is the most usable. The results of our study show that each of the evaluated systems has usability shortcomings and thus offers room for improvement. However, overall, the Sample Locator was rated as the tool with the highest usability. The analysis of the screen videos for this tool showed the fewest usability problems, and the questionnaire data showed the highest SUS score (SUS score: 77.03) for this tool. i2b2 is in second place, with a SUS score of 59.83. ATLAS was rated the worst; this system only achieved a SUS score of 27.81, and it was difficult to use from the perspective of the test subjects. The main reason for this difference in evaluation can be found in the complexity of the systems and the target group addressed by these tools: The Sample Locator is aimed at scientists and medical researchers who search for biosamples in academic biobanks. The selection of search criteria for samples is limited in the Sample Locator, so the Sample Locator is a very simple search tool. ATLAS is primarily designed for researchers and experts who need to assemble very complex cohort queries. Therefore, the variety of selection and input options is much higher, which also increases the complexity of operation. i2b2 offers a compromise between these systems. For the goal of the development in MIRACUM, it was asked which tool offers the best integration basis. The answer in this respect is that, of these three tools, the Sample Locator is the most user-friendly from the user’s point of view and is therefore considered the best basis for developing a tool for feasibility studies.

The second research question is related to the negative and positive design aspects of the three systems. The strengths of the Sample Locator were mainly its esthetic, minimalist design and the resulting clarity, the easy input of parameters, and the intuitive navigation. The main disadvantage was that it was not obvious in which logical way the parameters were linked after input. In addition, when entering the age of the donor, it was not clear why an input option for this was available in the two areas *Sample* and *Donor and Clinical Information* and what the difference of the selection option was. In addition, the Sample Locator had only limited functionality. For example, complex periods could not be defined or a concrete storage temperature could not be entered. For further development of the tool, it is recommended to address these usability problems.

i2b2 proved to be very intuitive to use with its *drag-and-drop* operating concept and offered a good and simple way of selecting parameters via the menu tree. However, even with this tool, the parameters were not always linked correctly, despite the short text-bright hints. One reason for this was that users would expect parameters to be linked with *AND* within a field and *OR* between fields. In fact, the opposite was true. Moreover, the display of the results proved to be unfavorable because test persons would not expect to have to select the *Refresh* option actively and repeatedly themselves to get an up-to-date display of the results. It should be noted, however, that the result display inherent in i2b2 is not used in the MIRACUM i2b2 context but in a connected project management tool. Thus, the result display is not a mere usability problem of the i2b2 feasibility tool. Due to the completeness and comparability between the tools, this issue was nevertheless considered in the assessment of i2b2.

From the point of view of the test subjects, ATLAS offered the greatest variety of input and selection options, but this made it difficult to keep relevant information and options clearly arranged and easily recognizable. It was also unclear to the test subjects why the selection of demographic parameters (eg, age and gender) followed a different selection principle than other parameters (eg, diagnoses or therapy). It was also not understandable why an exclusion criterion would have to be defined as a reverse inclusion in the *Inclusion Criteria* area. Most of the test subjects also failed to recognize how to start a search, as they did not link the *Generation* tab with a search option.

To the best of our knowledge, no study has previously compared the usability of query builders for feasibility studies. However, we were able to identify some studies that tested individual query builders with regard to their usability, which can be discussed with the partial results of our study:

- A usability study by Schüttler et al [29] with 27 participants rated a mock-up version during the development phase of the Sample Locator as intuitive and user-friendly. The mean SUS score of the Sample Locator was 80.4, indicating good usability. Our study showed a similarly high SUS score of 77.03, which also indicates good usability of this tool and supports the results of the study by Schüttler et al [29].
- A usability survey of the Criteria2Query tool, which performs queries in the ATLAS web application, revealed that almost half of the participants considered it difficult to perform the task of cohort definition (eg, identifying queryable eligibility concepts) [30]. Our study comes to a similar conclusion for the web tool ATLAS. The main reason for this is the complexity of the tool.
- A usability study of the EHR4CR (Electronic Health Records for Clinical Research) multisite patient count cohort system with 22 testers resulted in a SUS score of 55.83 (SD 15.37), indicating a low user satisfaction. The authors of the study stated that test subjects had problems, especially with complex queries [8]. We report similar results for all three tested query builders. In particular, queries that asked for *OR* or *NOT* links and a time constraint caused usability problems for the participants.
- An evaluation of a web application for cohort identification and data extraction revealed usability problems such as a missing *undo* function, which means that users could not directly return to the input mask to modify a query [31]. This was also one of the main problems reported with the i2b2 tool.

In summary, it can be said that individual studies come to a similar SUS assessment of the query tools and have reported similar operating problems in individual cases. However, this study, with its comparative design, represents the most comprehensive and systematic usability evaluation to date.

### Limitations

This usability study followed a comprehensive approach to compare the three query builders. However, some limitations must be considered when interpreting the results. Our results refer to a specific target group (researchers in Germany as laypersons or occasional users) and a specific context of use (feasibility queries of medium complexity regarding the general availability of patients or biosamples). The results can, therefore, not be transferred to other people (query experts and trained users who routinely use such tools) or a different objective (complex feasibility queries with the aim of finding very specific patients or biosamples). As other usability problems may arise for different contexts of use, our results are not generalizable. Furthermore, the selection of the three query builders was not based on an extensive analysis of the existing tools. Due to the limited timeframe of the project, this was dispensed with, and the focus was placed on tools that had already been used in other contexts in the project or were known from cooperation with other initiatives. Moreover, it was ensured that the tools are without exception, subject to an open source license, so that the most suitable tool can be used as a foundation for further development, if appropriate. A further limitation concerned the tasks, as the queries were designed for a test environment. To ensure functional comparability between the tools, the complexity of the tasks was based on the simplest tool. Although this did not allow all the functionalities of the other two tools to be fully exploited, care was taken to ensure that the tasks reflected real feasibility queries and thus covered all the required functions. The study procedure required each participant to evaluate all the three tools. Although the order of the test items was randomized to minimize bias due to learning effects, these cannot be completely avoided. However, owing to the strong differences in the basic operating concepts, we assume that such an effect is marginal. As the usability test was not conducted at only one location, it was not feasible to create one identical test scenario for all participants. The study was carried out by the participants for the most part at their workplace or, in rare cases, in their home office. However, we believe that this corresponds to a more realistic scenario than a laboratory setting, so that the insights gained are more informative. With regard to the tools, it should be mentioned that both ATLAS and the Sample Locator were provided in English only. Here, it must be taken into account that nonnative speakers may find some terms or options difficult to understand. In the case of this study, however, the majority of participants indicated that they had no difficulties with the English language (Table 2), so no bias due to comprehension deficits was expected. Finally, the relatively low number of participants (n=16) might be considered as a methodological weakness. During recruitment, the search for a population of suitable researchers and physicians with no profound knowledge of the tools was a bottleneck; therefore, we were unable to reach the targeted 30 subjects. However, this did not diminish the significance of the results. Kuric et al [32] showed that a sample size of approximately 15 participants yielded good results for the comparative usability evaluation of query builders; therefore, 16 participants were considered sufficient.

### Conclusions

This study provides data to develop a suitable basis for the selection of a harmonized tool for feasibility studies by concrete evaluation and comparison of the usability of three different types of query builders. The feedback of the participants during the usability test made it possible to identify user problems and positive design aspects of the individual tools and to compare them qualitatively. As a result, comparatively, the Sample Locator is the tool with the best usability, that is, the most positive ratings and the lowest number of usability problems. To create a harmonized tool, this tool is therefore considered the most suitable starting point. The Sample Locator outweighs the other tools in terms of the visual design of the user interface, clear and concise presentation of information, navigation, and presentation of results. For further development of the tool, there is a need to revise the display (visibility) of logical links, the provision of selection fields for diagnostic information, a clearer name or area placement of the *Donor Age* selection option, and a clearer presentation of the options for specifying a diagnostic period. Nevertheless, because of the current limitation of supporting only a small set of selection criteria and because, for example, no time constraints are possible, its scalability with respect to much more comprehensive sets of filter criteria (eg, with large possible value lists) and more complex Boolean expressions (including time constraints and dependencies) needs to be carefully considered. The results of our study provide valuable insights for researchers and developers of similar projects, whereby our methodological approach can be used as a blueprint. Our next step will be to apply the findings of this research to develop a harmonized feasibility platform. Although only laypersons were considered in this usability study, this tool could also be expanded in the future to include functions for experts to address an even broader user group. To obtain a holistic picture, experts who are already familiar with the field of feasibility queries will also be consulted.

## Appendix

Multimedia Appendix 1 Web-based questionnaire for usability evaluation (translated from German to English).

Multimedia Appendix 2 Identified usability problems with the highest severity rating (severity of 3 and 4) for the three query builders.

## Abbreviations

EHR4CR: Electronic Health Records for Clinical Research
FAU: Friedrich-Alexander-Universität Erlangen-Nürnberg
GBA: German Biobank Alliance
i2b2: Informatics for Integrating Biology and the Bedside
MII: Medical Informatics Initiative
MIRACUM: Medical Informatics in Research and Care in University Medicine
OHDSI: Observational Health Data Sciences and Informatics
OMOP: Observational Medical Outcomes Partnership
SUS: System Usability Scale
