# Correction: Evaluation of Three Feasibility Tools for Identifying Patient Data and Biospecimen Availability: Comparative Usability Study

**DOI:** 10.2196/33105

**Published:** 2021-10-08

**Authors:** Christina Schüttler, Hans-Ulrich Prokosch, Martin Sedlmayr, Brita Sedlmayr

**Affiliations:** 1 Chair of Medical Informatics Friedrich-Alexander-Universität Erlangen-Nürnberg Erlangen Germany; 2 Institute for Medical Informatics and Biometry Carl Gustav Carus Faculty of Medicine Technische Universität Dresden Dresden Germany

In “Evaluation of Three Feasibility Tools for Identifying Patient Data and Biospecimen Availability: Comparative Usability Study” (JMIR Med Inform 2021;9(7):e25531) the authors noted one error.

In the originally published manuscript, a funding code was not included in the “Acknowledgments” section. The following sentence has now been added to this section:

This work is additionally supported by the German Federal Ministry of Education and Research under the funding code 01EY1701.

The complete “Acknowledgments” section originally read as follows:

The authors would like to thank all participating MIRACUM locations—Dresden, Erlangen, Frankfurt am Main, Freiburg, Gießen, Greifswald, Magdeburg, and Mannheim. The authors would like to specially thank all scientists and researchers who participated in this study and provided a valuable insight into the usability of the different query builders through their *loud thoughts* and questionnaire evaluations. The authors would also like to thank Stefanie Schild (Erlangen), Renate Häuslschmid (Freiburg), and Preetha Moorthy (Mannheim) for their support in pretesting the tasks and questionnaires. This study was conducted as part of MIRACUM. MIRACUM is funded by the German Federal Ministry of Education and Research within the Medical Informatics Funding Scheme under the funding codes 01ZZ1801A and 01ZZ1801L.

The present work was performed in fulfillment of the requirements for obtaining the degree “Dr. rer. biol. hum.” from the Friedrich-Alexander-Universität Erlangen-Nürnberg (FAU) (CS).

This has been corrected to:

The authors would like to thank all participating MIRACUM locations—Dresden, Erlangen, Frankfurt am Main, Freiburg, Gießen, Greifswald, Magdeburg, and Mannheim. The authors would like to specially thank all scientists and researchers who participated in this study and provided a valuable insight into the usability of the different query builders through their *loud thoughts* and questionnaire evaluations. The authors would also like to thank Stefanie Schild (Erlangen), Renate Häuslschmid (Freiburg), and Preetha Moorthy (Mannheim) for their support in pretesting the tasks and questionnaires. This study was conducted as part of MIRACUM. MIRACUM is funded by the German Federal Ministry of Education and Research within the Medical Informatics Funding Scheme under the funding codes 01ZZ1801A and 01ZZ1801L. This work is additionally supported by the German Federal Ministry of Education and Research under the funding code 01EY1701.

The present work was performed in fulfillment of the requirements for obtaining the degree “Dr. rer. biol. hum.” from the Friedrich-Alexander-Universität Erlangen-Nürnberg (FAU) (CS).

The correction will appear in the online version of the paper on the JMIR Publications website on October 8, 2021, together with the publication of this correction notice. Because this was made after submission to PubMed, PubMed Central, and other full-text repositories, the corrected article has also been resubmitted to those repositories.

