# Supplementary material for: Evaluation of Three Feasibility Tools for Identifying Patient Data and Biospecimen Availability: Comparative Usability Study
Source: JMIR Med Inform. 2021 Jul 21;9(7):e25531. doi: 10.2196/25531 (PMC8339981; doi:10.2196/25531)
Supplement: Multimedia Appendix 1 [file medinform_v9i7e25531_app1.docx]

## Questionnaire Part A-C: Satisfaction and Usability

(to be filled in for each query builder; identical questions)

Task difficulty

How difficult were the tasks for you overall? Please indicate your assessment on the following scale: very easy/ easy/neither difficult nor easy/ difficult/very difficult

Satisfaction with the task

How satisfied were you with the query builder during task processing? Please indicate your agreement with each statement on the adjacent scale from "strongly disagree" to "strongly agree". (5-level rating scale: strongly disagree, disagree, neither agree nor disagree, agree, strongly agree)

- I am satisfied with the ease with which the tasks can be accomplished.
- I am satisfied with the time it takes to complete the tasks.
- I am satisfied with the functionality that is provided to complete the tasks.

Lack of functionality

If you have previously indicated that you strongly disagree/disagree/neither agree nor disagree with the functionality offered for the tasks: Which function(s) were you missing for the tasks? (free text)

Satisfaction

Please indicate your agreement with each statement on the adjacent scale from "strongly disagree" to "strongly agree". (5-level rating scale: strongly disagree, disagree, neither agree nor disagree, agree, strongly agree)

- The terms and designations used in the query builder (e.g. for the selection options, for patient characteristics) are immediately understandable to me.
- The query builder enables me to complete work steps (e.g. the selection of certain clinical/temporal parameters) in the order that seems to make the most sense to me.
- The results generated with the query builder are displayed or output in such a way that they meet my requirements (e.g. through clear grouping, an attractive visualization).
- It is immediately apparent to me which consequences my input in the query builder has.
- The query builder offers me the possibility to undo work steps if it is appropriate for my task completion.
- I found the navigation within the query builder easy.
- I found the information displayed in the query builder to be clear and concise.
- The user interface of the query builder is visually appealing.
- During my work with the query builder errors occurred (e.g. that options could not be combined; that exclusion criteria did not work).
- I sometimes felt slowed down in my work speed by the query builder, e.g. by too long waiting times.

System Usability Scale (SUS)

Please indicate your agreement with each statement on the adjacent scale from "strongly disagree" to "strongly agree". (5-level rating scale: strongly disagree, disagree, neither agree nor disagree, agree, strongly agree)

- I think that I would like to use this query builder frequently.
- I found this query builder unnecessarily complex.
- I thought this query builder was easy to use.
- I think that I would need the support of a technical person to be able to use this query builder.
- I found the various functions in this query builder were well integrated.
- I thought there was too much inconsistency in this query builder.
- I would imagine that most people would learn to use this query builder very quickly.
- I found this query builder very cumbersome to use.
- I felt very confident using this query builder.
- I needed to learn a lot of things before I could get going with this query builder.

## Questionnaire Part D: Final Rating and demographic information

Ranking of the tools

You have now tested the query builder i2b2, ATLAS and the Sample Locator. In your opinion, which of these three query builders is the most user-friendly and intuitive to use? Please put the query builders in an order according to the user-friendliness you estimate. 1st place means the most user-friendly and intuitive. (Options: i2b2/ATLAS/Sample Locator)

For the tool you rated "1^st^ place - most user-friendly":

- What did you particularly like about this tool?
- What improvements do you think still need to be made to the tool?
- Which functions of the 2^nd^ /3^rd^ placed tool would you recommend implementing for the 1^st^ place tool?

Questions about the person

- How old are you?: Age in years: (please specify)/no answer
- Which gender do you have?: male/female/other/no answer
- What is your mother tongue?: German/French/English/other: (please specify)/no answer
- Do you have any difficulties with the English language?: never/rarely/often/always/no answer
- To which professional group do you belong?: study manager/clinical researcher /scientific assistant/other: (please specify)/no answer
- How long have you been working in your current position?: work experience in years (please specify)/no answer
- How would you rate your experience with the retrieval of case numbers for clinical studies?: no-little experience/some experience/a lot of experience/no answer
- Have you used other similar systems in the past?: yes/no/no answer
- How would you rate your computer skills/knowledge?: low: I find many systems difficult to use/medium: I get along well with most systems/high: I have a lot of experience and am technically versed/no answer

Final remark

A questionnaire cannot address all aspects. This part of the questionnaire is reserved for your individual comments. Is there anything else you would like to draw our attention to? (free text)
