# Supplementary material for: Evaluation of Three Feasibility Tools for Identifying Patient Data and Biospecimen Availability: Comparative Usability Study
Source: JMIR Med Inform. 2021 Jul 21;9(7):e25531. doi: 10.2196/25531 (PMC8339981; doi:10.2196/25531)
Supplement: Multimedia Appendix 2 [file medinform_v9i7e25531_app2.docx]

# Identified usability problems with the highest severity rating (severity of 3 and 4) for the 3 query builder

## i2b2

| **Usability problem description (error taxonomy)** | **Example quote** | **Severity** | **Number of persons** | **Recommendation** |
| --- | --- | --- | --- | --- |
| The user cannot search for criteria (name/codes) via the "Find Terms" tab, because the search does not work/was not implemented.  (Usage problem: error of knowledge) | *Maybe I could just enter it here... He didn't find it. I can't select one. But this is bad. Maybe here?..It's not there either. So I don't know here yet how to find it. Apparently he finds nothing...Which is bad here, that I don't know if he finds nothing or if he doesn't search at all.* | 3 | 10 | implement a search option |
| The user does not know how the date can be set and which operating steps would have to be taken.  (Usage problem: error of knowledge) | *I don't really know now. So, I would then perhaps simply take the date of the patient admission, because I don't really know...although admission, it is not really the admission, but I want to know when the diagnosis was made. I don't really know where I can find that now. With diagnosis it is somehow not included.* | 3 | 7 | train the user/offer help |
| After entering the date using the option "Constrain Item by date range" the "OK" button does not work. (However, if another criterion is added to the fields, it will be displayed as entered).  (Functional problem: action blockade) | *Okay, no? I can't... or did I check the wrong one? Now I don't know if he accepted it or not.* | 3 | 6 | solve the functional problem |
| It is not clear to the user which meaning the different fields/groups have and how the criteria within and between the fields/groups are logically linked.  (Usage problem: error of knowledge) | *So what is "and" and what is "or"? ...That's really confusing, because I'm not sure whether this is "and" or "or".* | 3 | 5 | place the link notes more prominently (better in the upper group area); train the user/offer help |
| On the results page, the user expects to be able to edit the request after selecting the button "Edit Request“. However, all entries are deleted and the user is returned to the start page.  (Usage problem: error of judgment) | *Where is my request...Where has my input gone? And how do we find it now? Oh no, it is gone. How do I find it again now? ...Well, I would have thought that I could come back here and then work on it again when it says "edit request". But probably it doesn't, and I have to do it all over again.* | 3 | 4 | usage of a different button name ("Back to start screen" or similar) combined with a warning message to be confirmed that all entries will be deleted |
| It is not clear to the user how an exclusion criterion can be defined.  (Usage problem: error of knowledge) | *And apparently you can...Ah, wait a minute. So I would select all exclusion criteria. But you can't do that individually in a group. So if you click on it, everything is excluded.* | 3 | 3 | train the user/offer help |
| The user assumes that after executing the query (Button "Run Query") he has an opportunity to edit the query. But this does not work.  (Usage problem: error of knowledge) | *Now I'll make a query how many there are. Aha, ...oh. Now I have been kicked out.* | 3 | 2 | warn the user with a request for confirmation to avoid this situation |
| On the results page the user does not see how to start a new query due to the non-self-explanatory button designations (request, search, project request).  (Usage problem: error of reasoning) | *What happens next? Let's go to the start page or... This is a bit difficult. Where do I have to go now? Request, search, project request...* | 3 | 2 | use button names that speak the language of the user |
| On the results page, the user expects to be able to process the request after selecting the "Back" button. However, all entries are deleted.  (Usage problem: error of judgment) | *And if I click on "back" here now, it's exactly the same problem: I can't get back to the query builder where I actually wanted to go.* | 3 | 1 | display a warning message to be confirmed that all entries will be deleted |

## ATLAS

| **Usability problem description (error taxonomy)** | **Example quote** | **Severity** | **Number of persons** | **Recommendation** |
| --- | --- | --- | --- | --- |
| In view of the variety of input/selection options, the input of criteria is very confusing for the user. The user cannot see how he would have to proceed or cannot remember the concrete input steps later.  (Usage problem: error of memory and forgetting) | *This is all too confusing, too complex./* *Now I can't do what I had before... I can't find what I had before because everything looks the same.* | 3 | 12 | reduction of the input/selection options to the required |
| For the user it is not obvious how to define a criterion as "exclusion".  (Usage problem: error of knowledge) | *Good question where exclusion criteria are now defined. Here (area "Inclusion Criteria" under button "Add criteria to group") not... Yes, it is not quite clear how to define an exclusion here. Not quite clear to me. Really not quite clear. Also the question how to display exclusion criteria correctly is not clear to me. So the next thing that would fit would be "Censoring Events".* | 3 | 8 | train the user/offer help; insert an exclude button directly at the top (field) level; offer a second area "Exclusion Criteria“ |
| It is not clear to the user how the search can be started/executed.  (Usage problem: error of knowledge) | *So, how do I search now? I don't see a "Search" button now...I hope my entries are not all gone... "Reporting" maybe? "Export"- Ok, now it shows my search input again. And how do I find the whole thing now?* | 3 | 8 | choose a name for the button "Generate" that the user understands (e.g. „execute query“/“search“ or similar) |
| The user is unsure whether the input of measured values is correct, because no units are displayed or no possibility is seen to enter them.  (Usage problem: error of judgment) | *It is noticeable that you cannot specify a unit. /So this is the only way to select it. I hope, he will take it over, because somehow there is no confirmation, that he accepts all this.* | 3 | 7 | offer a direct input possibility of units directly on top level/field level |
| It is not clear to the user what he would have to select in order to enter the measured value or what the operating steps for this would be.  (Usage problem: error of knowledge) | *And here it is already difficult now, because I cannot enter it at all. So at least I can't find it at first sight./ And what I said before: I do not know where to click now. /How could that work now? Well, I can't solve that.* | 3 | 7 | train the user/offer help |
| The user does not understand why the "Save" button is disabled/greyed out (does not work if no "New Cohort Definition" has been entered or a search is running) and he cannot save.  (Usage problem: error of knowledge) | *This is here: the grayed button...“You must save.“- Well, does it also say how to save? This is the " Save"... It does not work. This is gray. Why?* | 3 | 7 | train the user; use clearer, constructive solution descriptions |
| The user is unsure whether the search will still produce a result, because the search is ongoing and no change in the search status (remains at "pending") is visible.  (Usage problem: error of judgment) | *The problem here is also that you don't see how long it takes. So I pressed the two buttons about - I would guess - 8 hours...6 hours? something like that, and it still works. And that's very difficult for a user to know, especially if you are not familiar with the program, how long does it take or does it work?* | 3 | 6 | insert a feedback how long the search will take or define a fixed time interval, how long the search may take at most |
| The user does not know how to start the search because the "Generation" buttons are grayed out.  (Usage problem: error of judgment) | *Generation... "Initial event is not set or changes are not saved"- but what is an initial event? Can I use the...? No - I cannot run it either. Something is still missing. But what is the initial event? So here I don't get any further at this point.* | 3 | 4 | use messages in clear language on how to solve the problem and offer a constructive solution |
| The user confuses the option "Observation Period" with "days after event index date".  (Usage problem: error of knowledge) | *365 days before or after event. This is a bit unclear to me.* | 3 | 3 | train the user/offer help |
| The user cannot see whether the text input fields for naming the "Inclusion Criteria" already represent the search or only the criteria can be named.  (Usage problem: error of knowledge) | *I would just try it over the text now. But whether this is really entered as a criterion I cannot say. So what I'm not really sure about right now is whether I'm actually asking for something or whether I'm creating a new search. Because if I enter a query here, I find it a bit strange that it should just come as free text. And that would be extremely error-prone. But if it's actually the direct input of a query, which I'm doing here, then I would actually expect selection options to be displayed. So I'm not really sure if I'm in the right function at all.* | 3 | 2 | train the user/offer help |
| It is not obvious to the user whether a new criterion field has to be created for each criterion (via the button "New criteria") or whether subfields should be created within a criterion (within a "New criteria").  (Usage problem: error of knowledge) | *The question is, do I have to put all this into one area or do I have to create four fields here (refers to the button "New inclusion criteria")...or all into one? I don't know right now.* | 3 | 2 | train the user/offer help |
| It is not clear to the user how a diagnosis date can be set.  (Usage problem: error of knowledge) | *And it would be nice if there was a "Condition Start Range" or something like that. But I don't think there is.* | 3 | 2 | train the user/offer help |
| The user cannot find an option/button to save the entered query.  (Usage problem: error of ecognition) | *How and where to save? I don't know. Where is this saved here? I don't know. I don't know where the task is saved. I can't save it any other way.* | 3 | 2 | place the button in the right, lower part of the screen (keep to conventions) |
| For the user, buttons are not always recognizable as such. In addition, the buttons are not uniformly designed (visually and functionally).  (Usage problem: error of ecognition) | *Here (refers to the blue link "add additional constraint") I'm not quite sure that this is another button. But the button (refers to the button "index start date") is basically a drop down menu. If you click on it something changes. Somehow this is not quite ideal.* | 3 | 1 | design buttons and selection fields visually and functionally in a uniform manner |
| The arrangement of the buttons (e.g. "Add criteria to group", "Add attribute") is confusing for the user.  (Usage problem: error of memory and forgetting) | *Well, the arrangement of the buttons is always confusing.* | 3 | 1 | place buttons in a way that confusion can be avoided |
| The user is confused because the operating concept is not consistent throughout: Different steps must be taken from the operating logic to select the "Diagnosis" criterion than to select the "Gender" criterion.  (Usage problem: error of habit) | *In my view, this is not implemented consistently. So here (area "Gender") you can get to the attribute via "Add attribute". And here (area "Condition Occurence") there is something else and you have to import a "Concept Set", which actually brings you to a search window and not to the...---mhm. Well.* | 3 | 1 | design the operating sequence for the selection of criteria in a consistent manner |
| The user is unsure whether entered inclusion criteria are linked with "AND".  (Usage problem: error of knowledge) | *If I now add a "Condition Occurence" here next, it will probably be linked with "AND". And here (related to the button "New inclusion criteria") linked with an "OR". But this I can only guess.* | 3 | 1 | train the user/offer help; clearly indicate the type of logical link |
| The user feels that the process of selecting a "Concept Set" is too complicated.  (Usage problem: error of memory and forgetting) | *Again the complicated field. Probably you have to search here again.* | 3 | 1 | reduce the input/selection options to the required |
| It is not clear to the user how to select the specific drug "Temozolomide" after selecting the option "Add Drug Exposure".  (Usage problem: error of knowledge) | *Yes, but why? But I already have "drug exposure"? Or do I have to select "condition occurence"?* | 3 | 1 | structure the user interface more clearly and reduce the input/selection options to the most essential; train the user/offer help |
| It is not clear to the user whether the diagnosis date entered refers to all criteria or only to the diagnosis "hypertensive disease".  (Usage problem: error of knowledge) | *Now I don't know if this date refers to this, the "hypertensive disease", or to everything. (HUC4)* | 3 | 1 | train the user/offer help |
| It is not clear to the user why the "New Cohort" button is diabled (this is the case if the previously entered cohort has not yet been saved).  (Usage problem: error of judgment) | *Why can't I click on a new one? Because I still have the query open somewhere?* | 3 | 1 | report in clear language how the problem can be solved and suggest a constructive solution |

## Sample Locator

| **Usability problem description (error taxonomy)** | **Example quote** | **Severity** | **Number of persons** | **Recommendation** |
| --- | --- | --- | --- | --- |
| It is not clear to the user what type of link exists or how the criteria can be correctly linked with "AND" or "OR".  (Usage problem: error of judgment) | *So, a bit unclearly honestly, whether this is "AND" or "OR". I mean, that's actually... that can't be "AND". But it is not clear.* | 4 | 11 | clearly indicate the type of logical link; offer help |
| It is not clear to the user how a diagnosis period can be specified or how this should be entered correctly.  (Usage problem: error of recognition) | *That means... I have to choose an area somehow. Whereby it is not quite clear to me whether I can do this now via "greater or equal" and "less or equal", regarding date entries. / That's now the question how I specify "in between".* | 4 | 5 | adhere to conventions for date entry; use concepts familiar to the user (e.g. date entry for online ticket bookings) |
| The user has no possibility to enter/select an exact storage temperature.  (Functional problem: alternative course of action) | *What are the options here? Now it should be minus 70 degrees. No, that doesn't even exist here... "Other storage temperature" doesn't make sense either.* | 3 | 7 | offer a possibility for direct input of the storage temperature |
| It is not clear to the user that "Age of Donor" and "Diagnosis Age" are two different concepts to be selected in two different areas (Sample and Donor/Clinical Information). For example, after entering the "Donor Ages" in the "Sample" section, the user expects to be able to enter the gender in this section (but can only be entered in the "Donor/Clinical Information" section).  (Usage problem: error of judgment) | *Where do we have the age again? Where was the age? Donor's age-No. "Diagnosis Age Donor"- What? What does "Diagnosis Age Donor" mean? So probably the age at diagnosis- Oh, there is the "Donor Age"! Why is that "Donor Age"? So somehow you can enter a "Donor Age" here again - with "Donor/Clinical Information" at the top and "Sample" at the bottom.* | 3 | 6 | possibly combine all options in one mask (without separate division into two areas); train the user, offer help |
| The user is confused because behind the button "Show Results per Biobank" he expects a display of the results per biobank and then tries different possibilities (e.g. by login of the institution) to have the results displayed after all.  (Usage problem: error of judgment) | *Okay, he didn't find anything here now. And the funny thing is, that on "Show"... yes, that I don't see anything here.* | 3 | 4 | use a clearer button description so that the user can see what is behind the option (e.g. "list of requested biobanks") |
| It is not clear to the user in which way the diagnosis should be entered so that the entry is correct and can be found.  (Usage problem: error of movement) | *Is there a selection format?- unfortunately not. /There is now also the question: Is it allowed to enter a fourth digit? Is there even a fourth digit? Which ICD classification should be used?* | 3 | 2 | offer a choice of options for the correct entry of the diagnosis |
